# A New Case of Hybrid Epidermoid and Apocrine Cyst

**DOI:** 10.3390/dermatopathology8030046

**Published:** 2021-09-01

**Authors:** Fulvia Serra, Gürkan Kaya

**Affiliations:** 1 Department of Pathology, University Hospital of Geneva, 1205 Geneva, Switzerland; 2 Department of Dermatology, University Hospital of Geneva, 1205 Geneva, Switzerland

**Keywords:** cutaneous cysts, hidrocystoma, hybrid epidermoid, apocrine cyst

## Abstract

We described a new case of a hybrid epidermoid and apocrine cyst, known to be a rare histopathological entity. The cyst was located in the axillary region and completely excised, without complication. The diagnosis was made at the histological analysis, where we found a cystic lesion in the dermis, lined with both epidermoid and apocrine epithelium.

## 1. Introduction

Hybrid cutaneous cysts are little known lesions that can be overlooked by the pathologist. Inside this small and curious family, the hybrid epidermoid and apocrine cyst is rare and its origin and clinical significance are unknown. Here, we present a new case found in the axillary region in a middle-aged woman with no other known comorbidity. We discuss the histopathological findings, the hypothesis of its origin and we underline the difficulties that can be encountered in reporting this peculiar entity. 

## 2. Case Report

**Figure 1:**
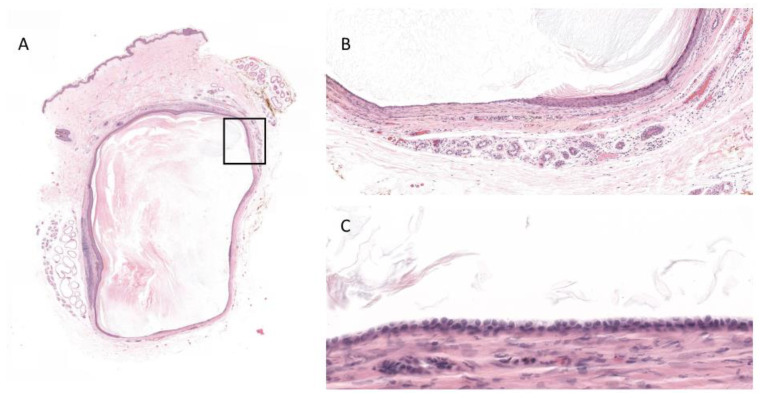
Histology. (**A**) HE ×10. The whole cyst. (**B**) HE Magnification ×20 (inset). Transition between epidermoid and apocrine epithelium. (**C**). HE ×40 Magnification. The apocrine epithelium.

A 45-year-old woman with a previous history of two cysts in the right axillary region (never excised), admitted to surgically remove one of the two, which measured 0.95 cm. To our knowledge, she does not have any prior disease. The cyst was removed without any complication and sent for analysis with the diagnosis of epidermoid cyst. The healing was uneventful. The histopathological analysis reveal a cystic lesion in the dermis (Figure 1). The cyst is lined in part by a squamous epithelium with an intact granular layer, sometimes atrophic, and in part by a distended apocrine epithelium. The passage between the two different epithelia is abrupt. We estimate that the apocrine component represents 30% of the epithelium. The cyst is filled with keratin lamellae. In proximity of the cyst’s wall, there are some apocrine and some sebaceous glands. No further exam was performed.

## 3. Discussion

In dermatopathology the cutaneous cysts are well known entities, but have been classified in different ways by many authors. In the literature, a simplified classification of cutaneous cysts, according to their origin in the folliculosebaceous unit and sweat glands, is proposed by Kaya and Saurat [1]. With this classification we can easily see that an epidermal cyst will have an infundibular origin, a tricholemmal cyst an isthmic origin, that a steatocystoma arise from a sebaceous duct, an eccrine/apocrine hidrocystoma from the glandular epithelial wall, and so on. We find this classification to be a useful tool also to discuss the hybrid cysts: once identified the different epithelia, we can easily refer to their origin. A hybrid cyst is in fact simply the combination of different types of epithelia found in the folliculosebaceous unit and sweat glands. The concept is introduced in a 1991 article on follicular hybrid cysts, by Requena et al., that defines the hybrid cyst as an entity arising from one component of the folliculosebaceous unit that can combine with others to form a large variety of follicular hybrid cysts, that show a distinctive two-component differentiation, with an abrupt transition between them [2]. It is in this peculiar family that we find the hybrid epidermoid and apocrine cyst, a combination of epidermoid epithelium with a hidrocystoma.

The epidermoid cyst is a well-known and common entity that is usually found on the face, neck, trunk and perineal area. The cyst is lined by a squamous cell epithelium that can be normal, atrophic or hyperplastic, but always with a well-defined and intact granular layer. It is filled with keratin lamellae.

The apocrine hidrocystoma is uncommon, it is rarely found in the axillary region (it usually arises in the head and neck area) and is generally a standalone lesion. Histologically, it appears as a cystic lesion, lined by a double layer of epithelial cells, the outer layer of flattened myoepithelial cells and the inner layer of cuboid columnar cells, with round or oval nuclei and decapitation secretion usually present. Cyst distention can cause flattening and atrophy of the epithelium, therefore sometimes it can be difficult to distinguish between an apocrine and an eccrine hidrocystoma. Since the apocrine and eccrine origins come from two ductal systems that are thought to be identical, some authors have proposed the alternative term of “ductal hidrocystoma”, to reflect the possible dual histogenesis [3].

To our knowledge, there are only few documented cases of hybrid epidermoid and apocrine cyst [4,5]. In a case report, Andersen et al., describe four cysts in four different patients: one on the eyelid, one on the lip and two on the nipple, measured between 0.5 and 0.6 cm [4]. The histopathological analysis shows the cysts lined with foci of cuboidal eosinophilic cells and decapitation secretion, alternated with a stratified squamous epithelium with an intact granular layer immediately adjacent. The luminas are filled with keratin lamellae.

Regarding the location, in a previous case report, Bourlond et al. [6] described two cases of erosive adenomatosis of the nipple, suggesting that an apocrine differentiation in a nipple’s cyst is not that uncommon as thought to be. Despite this argument, the more common location of the hybrid cysts with follicular and apocrine differentiation seems to for now to be the eyelid, like can sometimes happen in Moll gland cyst [3] and as described by Milman et al. [5]. It is noteworthy that our case is the first hybrid epidermoid and apocrine cyst to be found in the axillary region. We do not find this to be a surprising location for such a lesion, being aware of the abundance of apocrine glands in this area. That said, we have to consider that maybe the hybrid epidermoid and apocrine cyst is underdiagnosed: especially when the apocrine component is present in less than 50% of the epithelium, it can be missed depending on the embedding and cutting level.

As for the pathogenesis, it is still unknown. It is possible that this peculiar entity arises from the junction of squamous and glandular epithelium or that it is an epidermoid metaplasia of an apocrine hidrocystoma, as proposed by Andersen et al. Milman et al., found an immunoreactivity for carcinoembryonic antigen of the follicular component and for HMW-CK in the apocrine component, supporting the first mentioned theory above the second one. To them, an epidermoid metaplasia seems unlikely, because of the abrupt transition from an apocrine epithelium to a well-differentiated and mature squamous epithelium, with granular layer and dendritic cells. Of course, in our case, as previously mentioned, an eccrine origin, cannot be excluded. In addition, it ought to be underlined that the presence of sebaceous glands near the lesion can be related to a sebocystomatosis component.

Since Gardner cyst has epidermoid features merged with pilomatrixoma, this can also be regarded as a type of hybrid cyst. On that note, Chang et al. developed a retrospective study with 12 different entities of hybrid cyst, found in 71 patients, to study their clinical significance and possible association with Gardner syndrome, but they found no correlation [7]. Because of its rarity, there was no case of hybrid epidermoid and apocrine cyst in this series. In this regard, our patient was healthy, with no particular history of prior disease.

In conclusion, we find that the histopathological analysis of cystic lesions is sometimes too simplified by the pathologist and thus some interesting lesions can easily be missed. With a more scrupulous observation, we can probably be able to find more cases of hybrid cysts. A larger collection of hybrid epidermoid and apocrine cysts could be useful to study their origin (preferably with a well-documented immunohistological analysis) and eventually the possible association with genetic syndromes.
